# Pennsylvania State Veterinary Medical Association

**Published:** 1897-05

**Authors:** Leonard Pearson

**Affiliations:** Chairman


### SOCIETY PROCEEDINGS.

### PENNSYLVANIA STATE VETERINARY MEDICAL ASSOCIATION.

### REPORT OF THE COMMITTEE ON SANITARY SCIENCE AND POLICE.[1]

Mr. President and Gentlemen: As chairman of the Committee on Sanitary Science and Police I beg to submit to you the following report of the infectious diseases of animals as they have come under my observation during the past year and to make some statements in reference to the work of the State Live-stock Sanitary Board. The State Live-stock Sanitary Board has at this time been in operation for approximately one year. The State Veterinarian was appointed on January 1, 1896, and the Board was organized and its rules and methods were formulated shortly thereafter. It was not, however, until about this time last year that active work could be undertaken, so that my present report relates chiefly to observations that have been made since our last annual meeting.

The extent of the work that has been done during the past year and the rapidity with which it has grown indicate the manner in which the operations of the State Live-stock Sanitary Board have been accepted by the owners of animals and by the veterinary profession.

Inspections have been made in nearly every county of the State; in some counties they have been very numerous. It is gratifying to note that applications for assistance from the owners of animals afflicted with contagious diseases, particularly tuberculosis, are most numerous and are increasing in number most rapidly from those sections in which the most work has already been done.

A large number of diseases have been brought to the attention of the Live-stock Sanitary Board, among which I wish to mention the following as being of special interest and importance.

Cornstalk disease is an affection which has prevailed extensively in the corn-growing districts of the West, and particularly in Iowa, Nebraska, and Kansas, for a number of years, but very few outbreaks have been reported from the Eastern States, and so far as I know the disease has not been recognized in Pennsylvania until the present season. During the past few months several outbreaks of disease among cattle have been reported and investigated that were characterized by the following features: excitability, abdominal pain, disinclination to move, constipation, weakness, paralysis, and death. In every instance thus far it has been possible to determine that cattle thus afflicted were fed on cornstalks that were in a mouldy condition. In one outbreak that I looked into recently in Lebanon County it was found that six cattle had died. During the day they were kept in a yard that was littered with corn-fodder from a stack in an adjoining field. The fodder was very mouldy, and the stalks were thickly coated with a grayish mould near the base of the leaf. The yard was cleaned up and the use of this fodder discontinued, since which time there has been no recurrence of the disease.

[1] Read before the annual meeting at Philadelphia, March 2, 1897.

This disease has been the object of a special investigation conducted under the auspices of the United States Bureau of Animal Industry, but it has not yet been determined whether it is due to the infection of the animal by a fungus or bacterium that exists in the corn-fodder, or whether the disease is a toxæmia resulting from the ingestion of the chemical substances produced by the organisms that multiply in corn-fodder under certain conditions.

Dr. Edwin Hogg, of Kirkwood, Lancaster County, successfully treated six cows afflicted with this disease, while an equal number in the same herd died. His treatment consisted in administering Glauber's salt and the use of atropine subcutaneously. The paralyzed animals were raised in slings twice a day and kept up for a short period each time.

Post-mortem examination of animals dead of cornstalk disease usually reveals a negative condition. Sometimes there is an effusion of serum in the connective tissue about the kidneys, and the lungs may be somewhat congested or œdematous. The fact that the lesions are so slight tends to support the view that the disease is a poisoning due to the ingestion of the substances mentioned.

Abortion of cows is a disease that has occasioned great losses in many parts of the State, and particularly in the dairy districts. Many cases have been reported in which it recurs in herds from year to year, so that the profit from the herd is entirely destroyed, and in some instances the losses that result from this cause are ruinous. The disinfection of the premises and the disinfection of the genital passages and organs of the cows usually result in at least an amelioration of the condition. The subcutaneous injection of a weak solution of carbolic acid is also highly recommended by some who have tried it, and my own experience with this method of treatment has encouraged me somewhat. As yet it is not possible to explain how carbolic acid administered in this way and in such small quantities could have a beneficial action, but Dr. M. E. Conard, of West Grove, who has used it in a number of outbreaks, has great faith in its efficacy.

The cause of abortion is still undetermined, and this should be a fruitful field for investigation. So long as the cause of the disease remains unknown all measures directed against it are like fighting an enemy in ambush, but as soon as the cause is discovered and its characteristics are made known the sanitarian has decidedly the best of the contest.

Hog-cholera has prevailed extensively all over the United States during the past season. It is estimated that the loss in Iowa resulting from this disease alone has amounted to more than $10,000,000 during the past twelve months, and while other States have not suffered so excessively they have likewise lost extensively. Most of the outbreaks that have appeared in Pennsylvania have occurred as the result of the introduction of Western hogs for fattening. Stock-hogs from the stock-yards in Chicago and Buffalo have nearly all of them been exposed to hog-cholera, because the disease has prevailed to such an alarming extent that it is scarcely possible for a susceptible animal to pass through a stock-yard or to be shipped in undisinfected stock-cars without being exposed to the contagion of this affection. When once introduced into a farming district in this way hog-cholera is spread by the interchange of animals or objects that have been in contact with them. In some instances the spread of the disease has been hindered or prevented by quarantining infected herds and confining them strictly to the premises of their owners. Some of the outbreaks that have occurred during the past season have been characterized by the extreme rapidity with which they have spread and destroyed their victims.

In my last annual report, for the year 1896, I stated that the total losses from this cause would probably amount to $350,000. Subsequent observation and correspondence have convinced me that my estimate was too low, and that the losses amount to at least five or six hundred thousand dollars.

Since the work of the Live-stock Sanitary Board is wholly new and in a condition of development, active measures relating to the control of this disease have not been undertaken with the exception of quarantining some of the infected herds, and in that way protecting swine of the immediate vicinity, and the distribution of a bulletin entitled *Hog-cholera and Swine-plague,* issued by the Bureau of Animal Industry. It is hoped that more effective measures can be devised and inaugurated before long.

Osteoporosis has prevailed to an alarming extent, or at least a large number of cases have been brought to my attention, during the past year. It may be, however, that the increase is more apparent than real, but it is evident that a large number of horses are incapacitated from this cause, and a number of vague and indefinite lamenesses result from this disease. The cause of osteoporosis is still undetermined, and until we have more information on this point all attempts to cure or prevent the disease will be purely empirical and probably to a large extent unsuccessful.

Glanders has not prevailed extensively. It may be, however, that since the dangers of this disease are so thoroughly understood only a portion of the cases that are dealt with by veterinarians and local authorities are reported to the Live-stock Sanitary Board. During the past year twenty-six horses with glanders were destroyed.

It has been shown beyond doubt during the past season that many of the outbreaks reported as anthrax are beyond doubt red water, and it seems probable that there is not nearly as much anthrax in this State as was formerly supposed. However, the genuine disease has appeared in Pennsylvania, and the State Live-stock Sanitary Board will have prepared and ready for use some vaccine to be employed in preventing this disease whenever it is necessary to use it.

The investigation of rabies, made under the direction of the State Live-stock Sanitary Board, has included visits to and inspections in the districts in which the disease has appeared, the tracing of animals reported as rabid and animals bitten by them, the inoculation of rabbits from the brains of dogs and other animals reported as rabid, and the quarantining and destruction of dogs and cattle in the acute stage of rabies and when they have been bitten by a dog that was undoubtedly afflicted with this disease. These investigations have demonstrated conclusively that rabies actually exists, and during the past season to quite an alarming extent, among the domestic animals of the State. It has been remarked that some outbreaks of the disease are of an excessively virulent and others of a comparatively mild nature—that is, some dogs produce rabies in nearly every animal bitten by them, while others produce rabies in a comparatively small proportion of the animals bitten, and sometimes the disease appears in the paralytic or dumb form. Variations in the strength of the virus of this disease have been studied for a long time, and have been produced artificially in laboratories, and it has been observed that under certain conditions the disease becomes progressively milder until finally the virus loses its virulence to such a degree that it will not produce the characteristic symptoms of this disease, but by passing this attenuated virus through an animal of different species its virulence can be restored, and it has been suggested that rabies of dogs always has a tendency to become milder with each succeeding generation, and unless another animal, a cat, for instance, is bitten and in turn bites a dog, thus propagating the disease, it will in time become practically harmless. There are some places in this State where rabies is stationary and cases among dogs are of frequent occurrence, but of such a mild type as to cause but little apprehension. But occasionally a dog in the furious stages of the disease will appear and bite animals or people and cause much alarm in a large territory, and a number of cases of rabies often follow a raid of this sort. While all undue excitement should be avoided, it appears that there is no good purpose to be accomplished by hiding the facts connected with this disease, but it should be explained fully and freely that the existence of rabies constitutes a serious menace to communities in which it exists, but that all dogs that have fits, become excited, or bite, are not mad, and that rabies is a comparatively rare disease, the diagnosis of which is attended by some difficulty.

The greatest attention has been devoted during the past year to the subject of tuberculosis of cattle. The question as to how to deal with this disease in the most profitable and satisfactory manner has been a difficult one to solve, and it has received the earnest consideration of the State Live-stock Sanitary Board. A great number of possible methods have presented themselves and have been suggested, but most of them have been rejected on account of obstacles that are not surmountable at this time. The plan that is pursued at present is to examine herds wherever and whenever examinations are applied for, but only upon receipt of a formal application which contains an agreement upon the part of the owner to do all in his power to prevent the reintroduction or development of tuberculosis in his herd, and to observe the precautions and measures recommended by the State Live-stock Sanitary Board for this purpose. Upon the receipt of an application of this kind a tuberculin-test of the entire herd is ordered, and the animals that prove to be tuberculous are appraised and destroyed, although an alternative has been provided in accordance with the following rules: When herds are examined and tested at the request of the owner: (*a*) Cattle that present physical symptoms of tuberculosis must be destroyed, after an arrangement as to their value has been made that it is satisfactory to an authorized representative of the State Live-stock Sanitary Board and to the owner or his agent, or after the cattle have been regularly appraised. (*b*) Cattle that respond to the tuberculin-test, but do not present physical symptoms of tuberculosis, and in particular, have healthy udders, may be kept by their owners, subject to the following conditions: 1. The tuberculous cattle shall be marked with a suitable tag in the ear, or otherwise, and quarantined on certain defined premises. These cattle shall be kept apart from other cattle and in buildings and enclosures in which other cattle are not kept and are never allowed to go. Or, if it is impracticable to provide a separate building for the tuberculous cattle, they may be kept in a building in which other cattle are stabled; provided, they are separated from other cattle by a tight partition, the construction of which is satisfactory to a representative of this Board, which divides the building into two entirely distinct apartments with separate doors, separate accommodations for feeding and watering, and separate yards. 2. The calves from these quarantined cows may be raised, provided that they shall, immediately after birth, be removed from the premises in which tuberculous cattle are kept, and that they shall not be allowed to drink any of the milk from their dams or other quarantined cows, except after boiling or heating to 185° F. 3. The milk from cows so quarantined shall be used for no purpose whatever, except after it has been boiled or heated to 185° F. 4. Cattle quarantined as above may be slaughtered for beef; provided, that they shall be inspected at the time of slaughter by a competent inspector approved by this Board, and the flesh must be destroyed or may be sold for food, according to the judgment of such inspector; and provided further, that when this alternative is selected it shall be in lieu of indemnity from the State Live-stock Sanitary Board, and all claim for indemnity is thereby forfeited, except for such animals as are condemned by the meat-inspector, in which case the appraised value shall be paid. (*c*) If the owner of cattle that have responded to tuberculin-tests elects to accept the indemnity provided by the law, and so informs the authorized representative of the State Live-stock Sanitary Board, such cattle will be appraised, destroyed, and examined post mortem under the direction of a representative of the Board.

This arrangement has been made for the purpose of accommodating the owners of animals who do not wish to have them appraised and destroyed immediately after they are tested and found to be afflicted with tuberculosis. The conditions are such that it is not possible for tuberculosis to be spread by cattle kept in accordance with them, and, although they may not permit of the convenient utilization of tuberculous cows, it does not at this time seem possible to moderate them in any degree without endangering the live-stock interests.

One great difficulty that has confronted the Board in its work has been in relation to supplying a source to which farmers can go for cattle that they may be sure are free from tuberculosis, for the purpose of restocking herds from which tuberculous cattle have been removed. Cattle-buyers are recommended to purchase no cattle that they have not good reason to believe are free from tuberculosis, and since one cannot ascertain this point satisfactorily without the tuberculin-test, in these cases in which it is not possible to visit the herds from whence the cattle that are offered for sale come, and since it is not practicable to insist on a certificate of health based upon the application of the tuberculin-test in all cases, it has not always been possible for farmers to buy cows that they should be sure about. It has been suggested that this difficulty can be overcome by providing that all cattle coming into Pennsylvania from other States shall only be admitted upon presentation of evidence that they are entirely free from tuberculosis, and a bill has been introduced before the present Legislature that will establish this requirement. This will make it possible for farmers to purchase cattle that they can be sure about, and will be a long step in advance of our present position, and at the same time will not add to the expense that the State is already put to. About six thousand cattle have been tested with tuberculin under the direction of the State Live-stock Sanitary Board, and of these about one-fourth have been condemned; but, of course, this does not represent the actual percentage of tuberculosis among the cattle of our State, because inspections have only been made in herds that were supposed to be infected. However, many of those herds that were supposed to be infected have proved entirely free from tuberculosis. The percentage of tuberculosis in herds has varied from 1 to 100 per cent., and week before last a herd comprising about 175 was found to be tuberculous to the extent of 95 per cent. It has become perfectly evident that the percentage of tuberculosis in the herd depends principally upon the length of time that the infection has existed. In old cases of infection, particularly in breeding herds, the entire stock is sometimes saturated with the disease; whereas, if the infection is recent a comparatively small number of animals are found to be afflicted, perhaps not more than one or two. The sanitary conditions also determine to some extent the rapidity with which tuberculosis spreads in a herd. Notwithstanding the general views in regard to this point and my own preconceived opinions upon it, I have not been able to demonstrate to my own satisfaction just what influences are most favorable or most unfavorable for the rapid transmission of this disease. It is quite easy to deduce inferences on this subject based upon our knowledge of the disease, the vitality of the tubercle bacillus, etc. While these inferences may seem to be supported in some isolated cases, the careful observer will find many illustrations of exceptions to what he has supposed should be the rule. For example, tuberculosis has been known to spread with great rapidity among animals kept in well-ventilated stables and stables that were well lighted, and even out of doors, while the cattle were at pasture. Some observations made in Iowa and some made in Scotland show that tuberculosis can spread extensively in herds that are never stabled.

Since it has been shown conclusively that heredity has but little part in the transmission of tuberculosis, there has been a general tendency among writers on this subject to dwell upon the importance of the inherited predisposition to the disease. But the very careful and extensive work conducted in Denmark by Prof. Bang seems to show that after all this is a matter of minor consequence, and general observation indicates that contagion, actual exposure to cattle afflicted with tuberculosis is the thing that is the most important to guard against, and if this is guarded against thoroughly the most important precaution has been taken.

It is, however, exceedingly important that one should know the exact value of good sanitary conditions, and what sanitary arrangements are best adapted to prevent the rapid spread of tuberculosis in a herd. A great many diverse opinions prevail on this subject, and a great many cheap views on it are aired from time to time in the public prints, but there is a great lack of accurate information and demonstrated facts that would be of great value and should be supplied. The State Live-stock Sanitary Board has realized the need of further knowledge in reference to many of the diseases of animals, and has instituted a laboratory at the Veterinary Department of the University of Pennsylvania, wherein investigations can be made and where tuberculin, mallein, anthrax vaccine, and black-leg vaccine are prepared. Several investigations are now under way, and it is earnestly hoped that the facilities for this work can be increased in proportion to its importance, so that more of the many vague and perplexing questions that beset the veterinarian may be studied and more effective means for the prevention and cure of diseases among animals may be developed.

I wish to thank the veterinarians of this State for their indispensable and ever-ready assistance whenever they were called upon to aid in the work of the Live-Stock Sanitary Board; the kind and willing manner in which they have responded to all calls made upon them has been exceedingly gratifying, and without it the results attained would have been impossible.

The fact that the live-stock industry of Pennsylvania represents approximately $125,000,000, and suffers losses approximating $6,000,000, as a result of diseases that are probably preventable, indicates the importance of the work that is going on under the direction of the State Live-stock Board.

This work will unquestionably grow in importance and extent, and that it may grow in such a way as to yield the greatest return for the money expended it is to be hoped that provision can be made for more extensive researches that will lead to a better understanding of the best means for the suppression of these numerous diseases, and this can only be learned by a more careful study of their causes, their exact methods of transmission, the conditions that are favorable and unfavorable to them, etc.

LEONARD PEARSON, V.M.D.,
Chairman.

